# Metacognitive Training for Negative Symptoms (MCT-Minus) in schizophrenia: study protocol for a pragmatic randomized controlled trial

**DOI:** 10.1186/s13063-026-09879-x

**Published:** 2026-08-28

**Authors:** Linda Swanson, Benjamin Rask, Jonas Persson, Pascaline Graner, Johan Bengtsson, Robert Bodén, Martin Cernvall, Ulrika Sällström, Mårten J. Tyrberg, Steffen Moritz, Simon Cervenka

**Affiliations:** 1 Department of Medical Sciences, Psychiatry, Uppsala University, Uppsala, Sweden; 2 Centre for Clinical Research Sörmland, Uppsala University, Eskilstuna, Sweden; 3 Division of Psychology, Department of Clinical Psychology, Uppsala University, Uppsala, Sweden; 4 Centre for Clinical Research Västmanland, Uppsala University, Västerås, Sweden; 5 Department for Psychiatry and Psychotherapy, University Medical Center Hamburg-Eppendorf, Hamburg, Germany; 6 Centre for Psychiatry Research, Department of Clinical Neuroscience, Karolinska Institutet & Stockholm Health Care Services, Region Stockholm, Stockholm, Sweden

**Keywords:** Schizophrenia, Negative symptoms, Metacognitive training, Cognitive model, Default-Mode Network, Randomized controlled trial, Pragmatic study, Mixed design

## Abstract

**Background:**

Negative symptoms are experienced by up to 60% of the individuals with schizophrenia and represent a stronger indicator of current and future functioning than positive symptoms. These symptoms respond poorly to pharmacological and existing psychosocial interventions, highlighting the need for targeted treatments. Previous research suggests that defeatist performance beliefs, combined with metacognitive deficits, may contribute to negative symptoms. A recent pilot study, adapting the cognitive intervention metacognitive training (MCT) to address negative symptoms, found symptom reduction, primarily due to improved reflective functioning and reduced perceived stigma. The current study aims to further assess the efficacy of this intervention and to explore potential mechanisms of change in negative symptoms.

**Methods:**

Ninety participants aged 18 and older with a psychotic disorder will be recruited from psychiatric care settings in Sweden. Participants will be randomly assigned to either the intervention group (MCT-Minus) or a control group receiving supportive counselling (SC). Data will be collected at baseline, post-intervention, and at 12-week and 12-month follow-ups. Magnetic resonance imaging (MRI) will be performed at baseline and post-intervention. Exit interviews will be held after completing the intervention. The primary outcome is change in negative symptom, measured by the Clinical Assessment Interview for Negative Symptoms (CAINS), across all time points. Repeated measures ANCOVA will be used to evaluate primary outcomes. Additional analyses will address changes in positive symptoms and potential mechanisms of change, including metacognition and internalized stigma. Qualitative data from exit interviews will be analyzed using reflexive thematic analysis.

**Discussion:**

This study is designed to evaluate the efficacy of MCT-Minus compared to supportive counselling. Given the limited treatment options for negative symptoms in schizophrenia, the findings may have important clinical implications.

**Trial registration:**

This study was registered on the 6th of November 2023 on Clinical Trials with the trial registration number NCT06127004. Statistical analyses will be preregistered on the Open Science Framework.

**Supplementary Information:**

The online version contains supplementary material available at https://doi.org/10.1186/s13063-026-09879-x.

## Background

Negative symptoms in schizophrenia refer to a reduction in behaviors and functions related to pleasure, social engagement, and affective expression as outlined in the National Institute of Mental Health (NIMH) Research Domain Criteria. These symptoms affect up to 60% of individuals with schizophrenia, are typically present through the duration of illness, and are stronger indicators of current and future functioning than positive symptoms [19, 79, 86]. As negative symptoms respond poorly to medication [28] and existing psychological interventions [8], there is a clear rationale for developing interventions targeting negative symptoms of schizophrenia [85]. This is especially important as very few previous interventions have specifically targeted negative symptoms, or indeed, measured negative symptoms as a primary outcome or distinguished between different negative symptoms. A previous meta-analysis [74] that specifically focused on psychological treatments for negative symptoms found moderate effect sizes for cognitive behavior therapy (CBT; *N *= 6; Hedges g = − 0.46) and cognitive remediation therapy (CRT; *N *= 2; Hedges g = − 0.59), which was reduced to a small effect (Hedges g = − 0.24) when only randomized controlled trials were included. Hence, there is a need to develop new treatments. Importantly, while clinicians tend to prioritize positive symptoms as primary treatment targets, patients frequently emphasize the importance of addressing negative and depressive symptoms [65]. Previous research suggests that negative symptoms are largely independent of positive symptoms, depression, cognitive dysfunction, and disorganization [27]. The relationship between depression and negative symptoms is particularly complex, involving both shared (i.e., anhedonia, avolition, anergia) and distinct (i.e., suicidal ideation and pessimism in depression versus alogia and blunted affect in negative symptoms), features which must be considered in the assessment process to ensure that the patients do not fulfill criteria for ongoing depressive episode [44].

With regard to psychological mechanisms, Beck et al.’s [5] cognitive model posits that negative symptoms arise when individuals adopt coping strategies that involve “shutting down” cognitive–affective experiences. While this allows individuals to cope with overwhelming or aversive situations in the short term, it can lead to long-term reliance on negative symptoms such as social withdrawal, avolition, and diminished expression. The model suggests that negative symptoms might be caused and maintained by dysfunctional beliefs formed through repeated failures and setbacks; these may include underlying defeatist beliefs relating to task performance, leading to a behavioral pattern of passivity and avoidance as the individual exaggerate the limited availability of resources. This may be accompanied by low expectancies relating to social acceptance due to stigma, which may explain the social avoidance typically seen in negative symptoms. In addition, social distancing beliefs are also frequently seen in individuals with negative symptoms, perhaps as a defense mechanism. The authors also suggest that the symptoms might be explained through low expectancies for success, which interferes with goal-directed behavior. Hence, the individual overexaggerates the possibility of failure and becomes hypervigilant for criticism due to underlying core beliefs. Finally, the model suggests that negative symptoms might be due to beliefs about pleasure as individuals with schizophrenia often do not anticipate that activities will be pleasurable (i.e., anticipatory anhedonia), even though they seem to have an intact ability to experience pleasure when they engage in the activities. According to the cognitive model, these factors may collectively diminish the individual’s motivation in everyday life and reinforce the “shutting down” strategy.

Longitudinal studies have shown support for the cognitive model as defeatist performance attitudes and asocial beliefs are found to predict future negative symptoms [31, 53, 82]. Negative symptoms have also been found to be associated with low expectations of success [20], reduced self-efficacy [7], negative self-concepts [49], defeatist performance beliefs [15], and self-stigma [40]. Although traditionally seen as a phenomenon exclusive to schizophrenia, similar cognitive processes have been observed in other clinical populations [66] and in non-clinical samples, then typically conceptualized as part of a depressive state [73].

When individuals begin “shutting down,” their metacognitive processes are also affected. In contemporary research, metacognition is defined as ranging “from discrete processes involving noticing specific thoughts and feelings to more synthetic acts in which information is integrated into complex representations of the self and others” ([29] p. 171). Though deficits in metacognition are not exclusive to negative symptoms of psychotic disorders, several authors highlight the links between negative symptoms and metacognitive processes [32, 34, 39]. For instance, individuals who struggle to interpret and assign meaning to their own thoughts may find it harder to set goals and work towards them. Similarly, uncertainty about others’ intentions could cause discomfort and social avoidance [62]. Furthermore, greater severity of negative symptoms has been associated with more pronounced metacognitive difficulties [58], and such deficits have been shown to predict negative symptoms in both first-episode psychosis [4] as well as in chronic cases [54]. Metacognitive processes have also been found to mediate the relationship between cognitive functioning and negative symptoms, particularly for deficits in capacity to communicate internal states, defined as diminished expression. Collectively, these findings suggest that improving metacognitive capacity may contribute to the reduction of negative symptoms, supporting the rationale for a metacognitive intervention specifically targeting these symptoms. A metacognitive intervention focusing on negative symptoms might therefore be more effective than existing psychosocial interventions used to treat negative symptoms (e.g., CRT; [60] or CBT; [74]) which were primarily developed to target other symptom domains (e.g., neurocognition or positive symptoms/depression) rather than core metacognitive processes in negative symptoms [16].

Metacognitive training (MCT; [67, 68]), originally developed to address positive symptoms in schizophrenia, is based on the premise that cognitive biases contribute to the development and maintenance of psychotic symptoms, which can be alleviated by targeting underlying cognitive processes [72]. The aim of MCT is to enhance insight and provide practical strategies for managing distressing symptoms [77]. The intervention has been shown to reduce delusions [50] and positive symptoms [59, 71] while improving cognitive insight [9, 76]. Preliminary evidence also suggests effects on quality of life [63] and illness insight [52]. However, like CBT and CRT [74], the standard MCT has shown only to have a small, though statistically significant, effect on negative symptoms (Hedges g = 0.23), though it is important to acknowledge that the original intervention does not explicitly target negative symptoms [70]. MCT, which has been described as an amalgam of psychoeducation, CRT, and CBT [63], might be effective in reducing various dimensions of negative symptoms through its combined focus as previous research found that CR, but not CBT, improved reduced expression while CBT, but not CR, was efficacious in reducing amotivation [74].

To address this limitation, [81] adapted MCT by integrating interventions aimed at the cognitions proposed to contribute to the development and maintenance of negative symptoms in the cognitive model [5]. Importantly, the treatment explicitly targets the “shutting down” behavior characteristic of negative symptoms, a mechanism not systematically targeted in prior psychosocial interventions such as CR and generic CBT. In a preliminary study, Metacognitive Training for Negative Symptoms (MCT-Minus) demonstrated good feasibility, as evidenced by greater than expected attendance rate, reductions in negative symptoms, and positive feedback from both participants and clinical staff. Multilevel modelling analyses showed that improvements in depression, internalized stigma, and reflective functioning accounted for changes in negative symptoms. A follow-up analysis showed significant improvements, specifically on items of sociality and blunted affect [80].

Regarding the associated brain mechanisms, metacognition has been linked to default mode network functioning (DMN). Specifically, high metacognitive capacity was related to efficient DMN connectivity in early psychosis [25]. Further, increased network homogeneity within the DMN following MCT for positive symptoms in patients with schizophrenia compared to controls was observed [78], and regional homogeneity within DMN regions including the precuneus and medial prefrontal cortex predicted treatment response [78]. Taken together, these findings suggest that disrupted DMN function is associated with metacognitive deficits and that this network may mediate MCT response. Here, we seek to extend these findings to MCT targeting negative symptoms. Increased knowledge about the neurobiological processes involved in negative symptoms may aid in developing new, more effective psychosocial and pharmacological treatment options [26, 36].

### Aims and purpose

The purpose of the present study is to further evaluate the clinical efficacy of MCT-Minus for negative symptoms. In addition, the study seeks to explore potential psychological mechanisms underlying symptom change, specifically defeatist attitudes, reflective functioning, and internalized stigma. A further objective is to examine biological correlates of treatment response through functional and structural magnetic resonance imaging (MRI). Finally, the study aims to contribute to the existing evidence base by assessing whether changes in cognitive biases targeted by MCT-Minus are associated with improvements in broader conceptualizations of metacognition.

## Method

The study was pre-registered at Clinical Trials with ID NCT06127004. The statistical analyses will be preregistered on Open Science Framework. The SPIRIT reporting guidelines were used in the writing of this manuscript [18]. See Supplement 1 for the SPIRIT checklist.

### Study design

This multicenter, assessor blind RCT will compare 8 weeks of MCT-Minus with 8 weeks of supportive counselling (SC) in participants with psychotic disorders. The primary outcome is negative symptoms, as measured by the Clinical Assessment Interview for Negative Symptoms [6, 43], and secondary outcomes include reflective functioning, depression, overall functioning, stigma, quality of life, and dysfunctional attitudes. Data will be collected at baseline, post-intervention, and 12-week follow up, The Motivation and Pleasure-Self Report (MAP-SR; [17, 51]) and the Treatment Credibility Scale [10] will be administered mid-intervention. Participants in the MCT-Minus group will also undergo 12-month follow-up (Table 1). Magnetic resonance imaging (MRI), including rs-fMRI, will be performed pre and post intervention and at follow-up. As per standard clinical-trial safety monitoring, the trial will be paused or terminated should any serious adverse event plausibly related to the intervention occur.

**Table 1:** Schedule of enrolment, interventions and assessments

	**TRIAL PERIOD**													
	**Enrollment**	**Allocation**	**Post-allocation**											**Close-out**
**TIMEPOINT**	-t1	0	Week 1	W. 2	W. 3	W. 4	W. 5	W. 6	W. 7	W. 8	W. 9	W. 10	3 months follow-up	12 months follow-up
**ENROLMENT:**														
**Eligibility screen**	x													
**Informed consent**	x													
**Allocation**		x												
**INTERVENTIONS / COMPARATOR:**														
**MCT-Minus**														
**Supportive counselling**				x	x	x	x	x	x	x	x			
**ASSESSMENTS:**				x	x	x	x	x	x	x	x			
**CAINS, PANSS, ISMI-9, WHODAS, DAS, RFQ-8, CDSS, EQ5D.**			x									x	x	x
**TCS, MAP-SR**			x					x				x	x	x
**Qualitative interview**												x		
**MRI**			x									x		

### Participants

Clinical teams in the Swedish regions Sörmland, Västmanland, and Uppsala will be approached and asked to inform suitable participants about the study. Written informed consent will be obtained from the participants before any data collection or intervention. Inclusion criteria for participants are as follows: over the age of 18, unchanged psychotropic medication regimen during the past month, and diagnosed with schizophrenia, delusional disorder, non-affective psychosis, schizoaffective disorder, or unspecified non-organic psychosis. Exclusion criteria are as follows: evidence of severe organic brain dysfunction or a learning disability, difficulty with the Swedish language, visual and/or hearing impairment, and/or being unable or unwilling to provide written informed consent. In addition, for participants consenting to MRI, exclusion criteria include contraindications for this specific procedure (e.g., implanted or embedded ferromagnetic or electromagnetic objects that would present a risk during the scanning session).

### Study procedures

After inclusion, sociodemographic data including diagnosis, duration of illness, and current medication are collected from participants assisted by medical health records. Participants will be randomized (1:1) to either MCT-Minus or SC using permuted block randomization by a researcher not involved in assessments or delivering the intervention, where the primary investigator will assign participants to the allocation. The primary investigator stores information on randomization and allocation in a separate file, which is not shared with anyone. When baseline assessment is completed, the primary investigator reveals allocation to the research therapist. Thus, researchers performing assessments and the participants are blinded to the allocation, while research therapists and the primary investigator are not. Unblinding is not allowed during the trial; participants allocated to the control condition are unblinded after the 12 weeks follow-up as they are offered the active treatment. After the 12-week follow-up, participants originally allocated to supportive counselling will have completed their participation in the study, regardless of whether they accepted the offer of active treatment. Only participants originally allocated to MCT-Minus will be included in the 12-month follow-up. Consequently, data from the 12-month follow-up will be non-comparative.

Participants will receive intervention from research therapists with a basic diploma in cognitive behavioral therapy corresponding to 45 ECTS credits. All research therapists are trained in MCT-Minus as well as supportive counselling, allowing them to deliver both interventions in respective groups. All participants will receive regular care irrespective of condition, which includes medical management and psychosocial support with providers who are in collaboration with other social services. Exit interviews will be held after completing the intervention, where a standardized interview schedule (with open-ended questions) will be applied to minimize question variation while retaining flexibility to assess individual experiences [69].

During the pre- and post-assessment visits, participants will also undergo MRI at Uppsala University Hospital. Participants from other sites agreeing to MRI will be accompanied by research staff. The protocol includes anatomical (T1-weighted) imaging and rs-fMRI, which will assess changes in default mode network (DMN) connectivity after the active compared to control intervention. Additionally, diffusion weighted imaging (DWI) and magnetic resonance spectroscopy (MRS) will be used to relate the functional connectivity findings to structural connectivity and prefrontal glutamate levels.

### Power

We have used t/z-test, with a power of 0.85, and a significance level of 0.05, to calculate sample size to find a difference of 4 units on the CAINS as this difference has previously been shown to represent the Minimal Clinical Important Difference [47]. We will aim to recruit 45 in each group to allow for drop-out, group heterogeneity, and to conduct future analyses (e.g., the effect of medication) as a minimum of 41 participants in each group is needed to reach adequate power. At the midpoint of recruitment (January 2028, 48 months in), we will review inclusion rates against projection; if recruitment is meaningfully behind, we will intensify outreach to clinical teams at the participating sites. A formal contingency review will be conducted 18 months before the planned end of recruitment (July 2030, 78 months in): if fewer than 50 participants have been enrolled at that point, we will (i) extend the inclusion period by up to 24 months and (ii) approach additional outpatient units within the participating study sites. We continuously monitor and address factors associated with recruitment failure in clinical trials that have been identified in previous research, such as over optimistic recruitment estimates, overly narrow eligibility criteria, insufficient engagement and training of recruiters, limited initial funding, and high participant burden [13].

### Interventions

#### MCT-Minus

Metacognitive training for negative symptoms consists of eight 45–60 min sessions, delivered individually as there is evidence indicating that this approach may lead to stronger effect sizes than delivery in a group format [50]. The original MCT intervention was adapted to negative symptoms by incorporating psychoeducation and strategies to target the cognitions suggested by the cognitive model [5] to be implicated in the development and/or maintenance of negative symptoms (see Table 2). Although some of the strategies have traditionally been used to target positive symptoms, it is assumed that the same reasoning styles lead to negative symptoms through the dysfunctional cognitions discussed previously (e.g., jumping to conclusions regarding social rejection and a dysfunctional attribution style reinforcing social withdrawal). As described more in detail in [64], the modules in the original MCT were added based on previous research: attribution [14], jumping to conclusion [22], changing beliefs [30], to empathize [84], memory [23], mood [48], self-esteem [21], and stigma [46]. The first module, where negative symptoms as conceptualized in the cognitive model were introduced, was created specifically for MCT-Minus. The intervention material will be provided on a digital tablet. MCT-Minus is partly picture-based and provides structured modules with easy-to-read language, to reduce the cognitive load for the participants. The intervention will be discontinued at the request of a participant or in the case of worsening disease.

**Table 2:** Summary of the modules included in Metacognitive Training for Negative Symptoms [81]

Session	Description
1	Introduction to negative symptoms (developed for MCT Minus) Psychoeducation on negative symptoms and how certain unhelpful cognitions (e.g., asocial/defeatist beliefs) might lead to/maintain these combined with cognitive restructuring to challenge them
2	Self-esteem (unaltered from the additional modules of original MCT) Psychoeducation on self-esteem and how this might lead to/maintain negative symptoms combined with strategies (e.g., becoming aware of social comparison, “joy diary,” cognitive defusion, and physical distraction) to develop a more balanced view of one’s strengths and weaknesses
3	Jumping to conclusions (JTC; modified from original MCT) Psychoeducation on JTC and how this might lead to/maintain negative symptoms combined with strategies (e.g., considering multiple interpretations) to monitor and challenge these
4	Attribution style (modified from original MCT) Psychoeducation on how one-sided attribution styles might lead to/maintain negative symptoms combined with strategies (e.g., considering multiple factors) to develop a more multifactorial explanatory style
5	Cognitive difficulties (modified from original MCT) Psychoeducation on how cognitive difficulties in psychosis and false memories may lead to/maintain negative symptoms combined with strategies (e.g., critically evaluating memories and conclusions drawn from such memories) to counteract the impact of these
6	Social cognition (modified from original MCT) Psychoeducation on how difficulties understanding facial expressions might lead to/maintain negative symptoms combined with strategies (e.g., gaining knowledge from the person, environment/situation, self-observation, facial expression, and gut feeling) to prevent faulty conclusions
7	Mood (unaltered from original MCT) Psychoeducation on how depression may lead to/maintain negative symptoms combined with strategies (e.g., cognitive restructuring) to challenge these
8	Stigma (unaltered from the additional modules of original MCT) Psychoeducation on how stigma may lead to and maintain negative symptoms combined with strategies (e.g., educating others about mental illness) to counteract the impact of stigma on one’s life

#### Supportive counselling

According to Safer & Hugo [75], a credible comparison treatment omits the therapeutic components of the active intervention while retaining the common factors of psychotherapy (e.g., therapeutic alliance and therapeutic rationale), thereby allowing for control of these nonspecific effects. Brief supportive psychotherapy is a widely used active control condition in psychotherapy research and is described as a “bare-bones common-factors treatment” ([56, 57] p. 122). In the present study, a standardized treatment manual for supportive counselling (SC) was developed based on the principles of brief supportive psychotherapy (as described in [56, 57]). Visual materials are similarly employed during SC sessions to mirror the approach used in MCT-Minus. Therapists are instructed to use open-ended questions to explore participants’ feelings and experiences, encouraging individuals to find their own insights rather than relying on therapist-directed techniques or structured exercises. Importantly, the intervention does not include any components of MCT-Minus such as specific psychoeducation on cognitive bias or metacognitive techniques Table 3.

**Table 3:** Summary of the modules included in supportive counselling

Session	Description
1–2	General psychoeducation on negative symptoms. Presentation of the rationale: talking about feelings and how they affect the patient can improve mental well-being. Information on the focus of the intervention: feelings and reactions to life struggles. Open-ended questions on the patient’s current difficulties, reactions to them, and coping mechanisms
3	Conceptualization: summarizing the life struggles, reactions and coping mechanisms previously presented by the patient. Repetition of rationale
4–8	Open-ended questions on the patient’s current difficulties, reactions to them, and coping mechanisms

#### Adherence to intervention and safety measures

Therapist manuals and visual materials for both MCT-Minus and SC are highly structured and user-friendly, minimizing the risk of therapist drift. To support treatment fidelity, weekly digital meetings are held by the research team, offering therapists from all study sites supervision and peer discussion. In addition, all study therapists are required to participate in four annual workshops focused on MCT-Minus and SC, aimed at reinforcing adherence to the respective protocols. During these workshops, role-play sessions based on the intervention manuals will be conducted. The fidelity of intervention delivery will be evaluated by members of the project group using a structured rating scale, such as the Cognitive Therapy Rating Scale [90]. Following the role-play sessions, structured discussions will be held focusing on potential therapist drift and other issues related to adherence to the intervention manuals. Attendance will be recorded, and systematic documentation will be kept of workshop and supervision content, including key discussion points, questions raised, and any deviations from protocol.

Should any clinical risk be identified during recruitment, assessments, or intervention procedures, research staff are required to report this at the scheduled weekly meetings to facilitate prompt coordination and communication with the relevant clinical teams.

#### Assessments

As the RCT is blinded, data collection and intervention will not be conducted by the same staff. The study will use a combination of interviews and self-rated questionnaires to assess negative symptoms. The primary outcome measure will be the Clinical Assessment Interview for Negative Symptoms (CAINS; [6, 43]) as it has been developed to measure negative symptoms as defined by the NIMH consensus development conference (i.e., blunted affect, alogia, anhedonia, asociality and avolition; [45]. The CAINS replaced the Brief Negative Symptom Scale (BNSS; [42]) that was used in the original study by [81] as a Swedish version of the BNSS has not been validated. In addition, the Motivation and Pleasure Scale–Self-Report (MAP-SR; [17, 51]) and the Positive and Negative Syndrome Scale (PANSS; [41]) will be used to measure negative symptoms (with the PANSS five-factor model proposed by [88]. To assess daily function, the 12-item interviewer-administered version of the World Health Organization (WHO) Disability Assessment Schedule (WHODAS) 2.0 [83] will be used, which has been suggested to be a more reliable measure than the Global Assessment of Functioning (GAF; American Psychiatric Association [APA], [2]) for assessing a person's overall functioning [33].

To identify and measure psychological mechanisms of change, four measures will be used: Dysfunctional Attitudes Scale (DAS; [89]) to measure the severity of defeatist attitudes; the Reflective Functioning Questionnaire (RFQ-8; [24]) to assess metacognitive capacity; the Calgary Depression Scale for Schizophrenia (CDSS; [1]) to measure depression; and the Internalized Stigma of Mental Illness Scale-9 (ISMI-9; [38]) to measure stigma. In addition, the Treatment Credibility Scale [10], will be used to measure trust in treatment while the EuroQol 5 dimensions (EQ-5D; [12]) will be used to measure quality of life.

## Analysis

### Quantitative data

Data will be analyzed once we have recruited a sufficient number of participants. Statistical analysis to evaluate changes at pre-, post-, and follow-up and to identify and measure potential mechanisms of change for negative symptoms will be conducted using linear mixed models. The overall analytic strategy will be to conduct analyses according to the intention-to-treat principle. In case of missing data, a missing data analysis will be conducted and appropriate methods for handling missing data will be used depending on the analysis [3]. Specifically, participant data in relation to the group they were randomly assigned to and consequently preserves the prognostic balance in the sample. After randomizing participants in RCTs, analysis of data is not automatically preserved from biased conclusions [61]. Missing data and noncompliance might pose a risk from biased conclusions; for example, if entire participant data were to be excluded from the analysis due to missing data, the risk of a biased estimate of treatment effect increases [35]. Additionally, exploratory analyses will be performed using sociodemographic data such as diagnosis, duration of illness, and current medication.

Preprocessing of rs-fMRI data will be performed with fMRIPrep [RRID:SCR_016216] followed by modeling with the conn toolbox [RRID:SCR_009550] in Matlab. FMRIPrep will perform head-motion and slice-timing correction as well as distortion correction using fieldmaps before normalization to standard space and generation of confound regressors. Preprocessed images and regressors are imported into conn, where Gaussian smoothing is applied to the data before denoising using CompCor regressors (physiological noise), motion parameters (translation/rotation and their first-order derivatives), and a variable number of scrubbing regressors for outlier time-frames. After applying a band-pass filter to the time-series (0.008–0.09 Hz), atlas-based brain parcellations will be used to calculate ROI-to-ROI connectivity and a DMN mask to calculate network homogeneity. An edge/voxel-wise repeated-measures ANCOVA will be used to assess brain-wide connectivity changes and changes in network homogeneity (NH) following the intervention, with time and intervention as factors and controlling for age. PANSS/CAINS scores will be included as a covariate in additional analyses to assess their relationship with NH and connectivity changes within the active intervention group. For statistical inference, a cluster-based approach will be used with a cluster-forming threshold of *p* < 0.001, uncorrected and a cluster-level threshold of pFDR < 0.05.

Preprocessing and reconstruction of DWI data will be performed with QSIPrep, including denoising, distortion correction, and head motion correction, followed by the generation of fractional anisotropy maps and the generation of structural connectomes using tractography based on the same parcellation as for the fMRI data. MRS data will be preprocessed and modeled in Osprey, including alignment and averaging of individual spectra, fitting of the spectra using basis functions included with the software, coregistering to an anatomical reference for tissue segmentation within the MRS voxel to finally quantify glutamate with CSF-corrected water-scaled estimates.

### Qualitative data

Reflexive thematic analysis [11] will be used to analyze the qualitative data. The interview, as is common in implementation research, will be based on a semi-structured interview guide with questions that are inviting, accessible, and analyzable (see [37]). The purpose of the interview (which incorporates questions covering the individual’s perception of the intervention and potential changes post therapy) is to capture the views of the “stakeholders” (i.e., perspectives which will help to guide and inform the research due to the expertise and role these individuals have in the healthcare system). The interviews will be transcribed, and transcripts will be read multiple times for familiarity with the material and to generate an overview of the responses [55]. The recordings will then be analyzed with reflexive thematic analysis conducted according to a standard format (i.e., exploring the feasibility of the intervention and potential mechanisms of change). Themes will be developed, labelled, and reviewed to ensure that they are representative of the data set. This analysis will be undertaken by a PhD-student (BR) and discussed with other research collaborators.

### Data management

Personal data is processed according to the European Union’s General Data Protection Regulation for research purposes, based on the legal basis of public interest. Initially, data is safely stored in a secure archive within the health-care system to which the principal investigator has access. REDCap, a web application for building and managing online surveys and databases, is to be implemented during the second half of 2025. Personal data will be pseudonymized, meaning names and social security numbers are removed and instead linked to a code. The code key and information on allocation are stored separately from the register. Only the principal investigator (LS) and the research coordinator have access to the code key. Personal data will be treated so that unauthorized persons cannot access them. When publishing research results, no individual's identity will be disclosed.

## Discussion

As the main aim, this study will add to the existing evidence base on the treatment of negative symptoms by examining if the promising results seen in the feasibility study of MCT-Minus [81] can be extended in a RCT with a control group and blinded assessments. The additional aims will serve to expand the knowledge on negative symptoms and its maintaining factors, including mechanisms of change, which is critical for further developing effective psychosocial and pharmacological treatments.

### Strengths

There are also several strengths with the design of the current study. The inclusion and exclusion criteria will ensure high ecological validity, by allowing participants with difficulties that the psychosis population often present, for example cognitive impairment, drug abuse and personality disorders. Furthermore, the study includes both qualitative and quantitative data, which enables deeper analyses and will contribute to a broader understanding of negative symptoms in psychosis, and subsequently, increase understanding of treating these symptoms.

### Weaknesses

Common negative symptoms such as avolition (i.e., lack of motivation) may contribute to difficulties with participation. Participants may not be motivated to begin or continue, resulting in premature drop-out from the study. Also, individuals with psychosis may have difficulties with compliance to prescribed antipsychotic medication due to poor insight, side effects, and/or cognitive difficulties [87], factors which could be applicable to psychosocial interventions as well. Although participants may understand the benefits of the intervention, anticipatory anhedonia might influence their ability to further motivate themselves. The participation in the study itself might also be experienced as distressing for some participants. Furthermore, it might be difficult to establish a therapeutic alliance with the participants due to positive (e.g., suspicion) or negative (e.g., asociality) symptoms. However, all research staff in the current study have clinical experience with people who suffer from complex psychiatric difficulties. Importantly, it will be necessary to liaise closely with healthcare providers to get assistance in recruiting participants and maintaining motivation as well as being flexible in terms of when and where to meet the participants (while still following local protocols regarding safety).

### Trial status

**Fig. 1:**
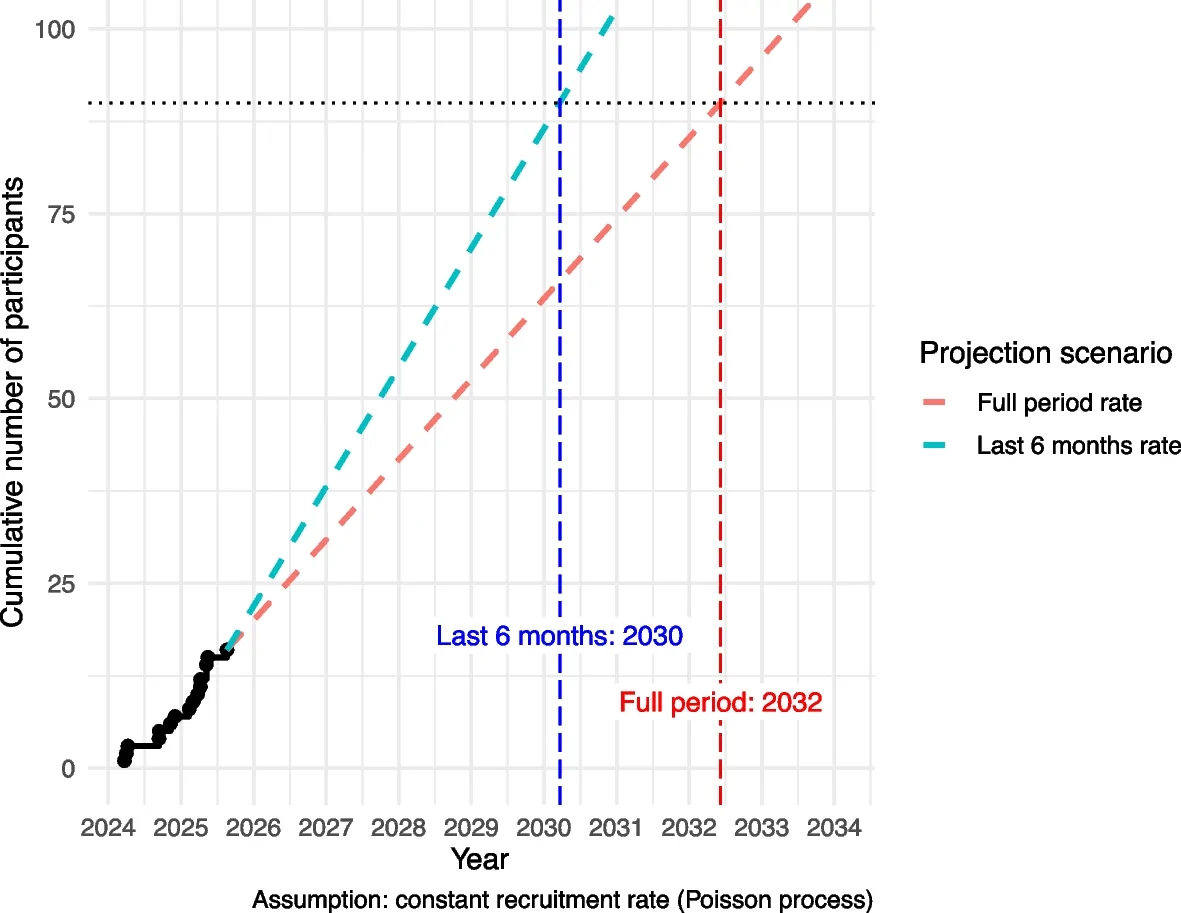
Recruitment projection to 90 participants using full-period and last 6-month rate

Recruitment started in January 2024. The current protocol is version 6 of 6–5–2026. Currently (6th of May 2026), we included 20 patients. In 2025, the number of outpatient units within our existing study sites that serve as recruitment sources increased from 3 to 6. Patient recruitment is estimated to be completed in January 2030 (Fig. 1).

## Supplementary Information


Supplementary Material 1.

## Data Availability

The data will be shared with other researchers in accordance with local regulations on data handling.

## Abbreviations

APA: American Psychiatric Association
BNSS: Brief Negative Symptom Scale
CAINS: Clinical Assessment Interview for Negative Symptoms
CBT: Cognitive Behavioural Therapy
CDSS: Calgary Depression Scale for Schizophrenia
CR: Cognitive Remediation
DAS: Dysfunctional Attitudes Scale
DMN: Default-Mode Network
DWI: Diffusion Weighted Imaging
EQ5D: EuroQol 5 dimensions
GAF: Global Assessment of Functioning
ISMI-9: Internalized Stigma of Mental Illness Scale-9
MAP-SR: Motivation and Pleasure Scale–Self-Report
MCT: Metacognitive training
MCT-Minus: Metacognitive Training for Negative Symptoms
MRI: Magnetic resonance imaging
MRS: Magnetic resonance spectroscopy
NH: Network homogeneity
NIMH: National Institute of Mental Health
PANSS: Positive and Negative Syndrome Scale
RFQ: Reflective Functioning Questionnaire
Rs-fMRI: Resting-state Functional Magnetic Resonance Imaging
SC: Supportive counselling
TCS: Treatment Credibility Scale
WHODAS: World Health Organization Disability Assessment Schedule 2.0
